# The Problem and Potential of TMS' Infinite Parameter Space: A Targeted Review and Road Map Forward

**DOI:** 10.3389/fpsyt.2022.867091

**Published:** 2022-05-10

**Authors:** Kevin A. Caulfield, Joshua C. Brown

**Affiliations:** ^1^Department of Psychiatry, Medical University of South Carolina, Charleston, SC, United States; ^2^Departments of Psychiatry and Neurology, Brown University Medical School, Providence, RI, United States

**Keywords:** repetitive transcranial magnetic stimulation, theta burst stimulation, parameter optimization, resting state fMRI, synchronized rTMS-EEG, synchronized TMS, inverted U-shaped curve, dose-response curve

## Abstract

**Background:**

Repetitive transcranial magnetic stimulation (rTMS) is a non-invasive, effective, and FDA-approved brain stimulation method. However, rTMS parameter selection remains largely unexplored, with great potential for optimization. In this review, we highlight key studies underlying next generation rTMS therapies, particularly focusing on: (1) rTMS Parameters, (2) rTMS Target Engagement, (3) rTMS Interactions with Endogenous Brain Activity, and (4) Heritable Predisposition to Brain Stimulation Treatments.

**Methods:**

We performed a targeted review of pre-clinical and clinical rTMS studies.

**Results:**

Current evidence suggests that rTMS pattern, intensity, frequency, train duration, intertrain interval, intersession interval, pulse and session number, pulse width, and pulse shape can alter motor excitability, long term potentiation (LTP)-like facilitation, and clinical antidepressant response. Additionally, an emerging theme is how endogenous brain state impacts rTMS response. Researchers have used resting state functional magnetic resonance imaging (rsfMRI) analyses to identify personalized rTMS targets. Electroencephalography (EEG) may measure endogenous alpha rhythms that preferentially respond to personalized stimulation frequencies, or in closed-loop EEG, may be synchronized with endogenous oscillations and even phase to optimize response. Lastly, neuroimaging and genotyping have identified individual predispositions that may underlie rTMS efficacy.

**Conclusions:**

We envision next generation rTMS will be delivered using optimized stimulation parameters to rsfMRI-determined targets at intensities determined by energy delivered to the cortex, and frequency personalized and synchronized to endogenous alpha-rhythms. Further research is needed to define the dose-response curve of each parameter on plasticity and clinical response at the group level, to determine how these parameters interact, and to ultimately personalize these parameters.

## Introduction

**Figure 1:**
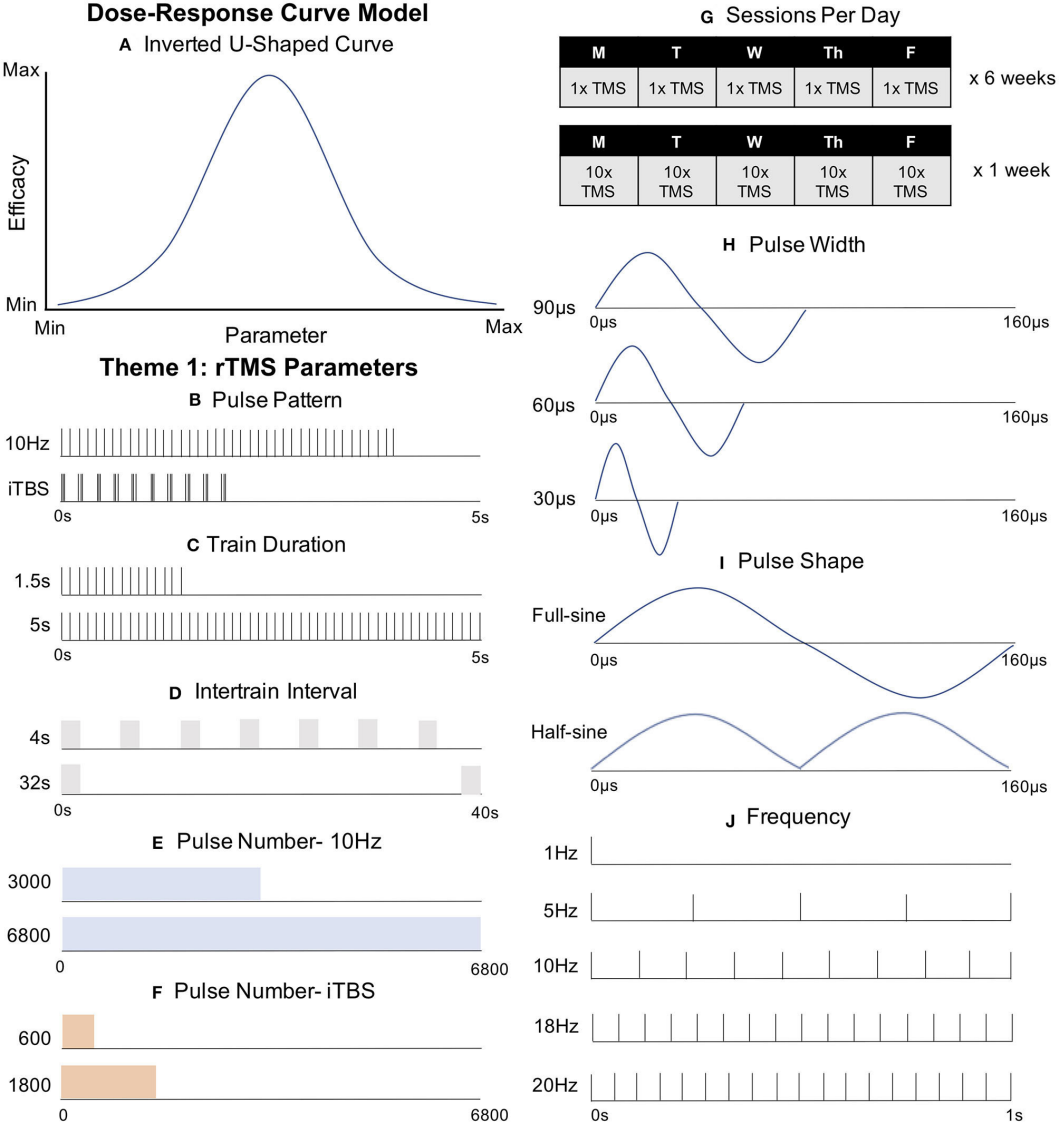
Key rTMS Parameters Guiding the Development of Next Generation rTMS Therapies. **(A)** Dose-Response Curve Model. Some parameters follow an inverted U-shaped curve, with peak efficacy in the middle. **(B)** Pulse Pattern. Intermittent theta burst stimulation (iTBS) has been FDA-cleared as a clinically non-inferior, but more efficient, form of rTMS compared to conventional 10 Hz stimulation. **(C)** Train Duration. Trains of 1.5 s at 10 Hz have produced the canonical excitatory effect while 5 s trains at 10 Hz produced an opposite inhibited effect. **(D)** Intertrain Interval (ITI). Decreased ITI has drastically reduced intracortical inhibition without changing corticospinal excitability or clinical depression outcomes. **(E)** Pulse Number-10 Hz. 6,800 pulses of 10 Hz rTMS did not improve clinical outcomes compared with conventional 3,000 pulse 10 Hz rTMS. **(F)** Pulse Number- iTBS. Doubling pulse number (1,200) produced inhibitory effects, opposing the excitation from the FDA-cleared 600 pulse protocol. **(G)** Sessions Per Day. Relative to conventional rTMS (*top*) “Accelerated rTMS” (applying more than one session per day, *bottom*), may produce a more rapid and effective clinical response. **(H)** Pulse Width. Longer pulse widths may produce more efficient cortical activation. **(I)** Pulse Shape. Full-sine (biphasic) waveforms appear to produce stronger stimulation than single or summated half-sine (monophasic) pulses. **(J)** Frequency. Despite extensive clinical investigation, various stimulation frequencies (including 20, 18, 10, and 5 Hz to the left DLPFC and 1 Hz to the right DLPFC) have not revealed a superior frequency at the group level.

Repetitive transcranial magnetic stimulation (rTMS) is a non-invasive, effective and FDA-approved brain stimulation treatment for treatment resistant depression (TRD) (1), obsessive compulsive disorder (OCD) (2), and smoking cessation (3). While conventional once-daily rTMS elicits remission in ~30% of TRD patients in a naturalistic setting (4), parameter selection remains largely unexplored, in part due to the infinite combination of possibilities. In this narrative mini-review, we highlight key studies demonstrating the potential impact that parameter selection can have on brain plasticity and clinical response, specifically focusing on: (**1) rTMS Stimulation Parameters** (i.e., Pattern, Intensity, Frequency, Train Duration, Intertrain and Intersession Intervals, Pulse and Session Number, Pulse Width, and Pulse Shape); (**2) rTMS Target Engagement**; (**3) rTMS Interactions with Endogenous Brain Activity**; and (**4) Heritable Predisposition to Brain Stimulation Treatments**. Theme 1 involves rTMS parameters and how they affect the brain; in contrast, Themes 2–4 highlight how underlying brain state affects stimulation efficacy. Understanding and applying optimized rTMS parameters holds enormous potential to improve next generation rTMS therapies across brain disorders, particularly as multiple variables do not simply produce better results with more or higher magnitude stimulation, but rather, appear to follow an inverted U-shaped curve with peak efficacy in the middle (Figure 1A).

### Parameter Theme 1: How Do RTMS Parameters Impact Brain Activity and Therapeutic Response?

#### Pulse Pattern

The most notable and widely adopted parameter change to date is pulse pattern. The only FDA-cleared form to date, intermittent theta burst stimulation (iTBS), typically delivers 600 pulses of rTMS in 5 Hz triplet bursts of 50 Hz pulses in sessions that take ~3 min. These parameters are based on traditional protocols shown to induce long term potentiation (LTP)-like facilitation, and are designed to emulate endogenous hippocampal activity (5) (Figure 1B). While iTBS is clearly faster than conventional 10 Hz rTMS protocols, it is unclear whether iTBS has greater, similar, or inferior efficacy compared to 10 Hz with mixed findings to date. In a motor evoked potential (MEP) study in healthy adults, Di Lazzaro et al. (6) found that iTBS increased MEP amplitude significantly more than 5 Hz rTMS. Similarly, Zhao et al. (7) found that iTBS produced significantly greater reductions in negative schizophrenia symptoms than 10, 20 Hz, or sham stimulation. Other studies have found similar results between theta burst and conventional rTMS protocols. In depression, a large non-inferior clinical trial found that iTBS produced nearly identical response rates as conventional 10 Hz rTMS (8). Tsai et al. (9) conducted a randomized controlled trial comparing 5 Hz rTMS and iTBS for post-stroke cognitive impairment, finding that both were effective in treatment certain symptom clusters.

While iTBS is faster to administer and could have superior or similar efficacy to conventional rTMS protocols, other studies have found that conventional rTMS protocols produce superior results, particularly in comorbid post-traumatic stress disorder (PTSD) and major depressive disorder (MDD). Whereas, Philip et al. (10) found that iTBS effectively treated PTSD acutely, and with durable effects assessed out to 1 year post-treatment (11), a retrospective chart review in patients with comorbid PTSD and major depression revealed that 5 Hz stimulation produced superior reductions in PTSD and MDD symptoms than iTBS (12). These data suggest that iTBS may not be the answer in all cases, and may even work through a different cellular mechanism, as 10 Hz rTMS and iTBS produced opposing MEP results in healthy controls when combined with NMDA receptor agonists (13–16).

#### Train Duration

The most commonly used iTBS protocol is based on the seminal findings by Huang et al. (5), who found that twenty 2 s trains (30 pulses per train) with an 8 s intertrain interval (ITI) produced facilitation for 15 min. It is worth noting that a single 2 s train could produce facilitation for up to 15 s, but a 5 s train caused inhibition at 10 s (7), suggesting that the optimal amount of stimulation is consistent with an inverted U-shaped curve “sweet spot” (Figure 1A).

The same principle appears to also apply to traditional rTMS, as Jung et al. (17) found that 1.5 s trains of 10 Hz rTMS produced the canonical excitatory high-frequency effect, while 5 s trains inhibited MEP amplitudes (Figure 1C). Interestingly, another group used 8 s trains, also for 20 min at 10 Hz, and observed increased facilitation (18). While increasing the train duration also increases the overall number of pulses, it may hint at a non-inverted U-shaped curve, at least within certain limits. Despite these insightful studies, we still do not know where this theoretical U-curve rises and falls, or where it peaks. Further delineation promises to fine-tune current protocols.

#### Intertrain Interval

Intertrain interval (ITI) refers to the time between trains of rTMS, and to date, has largely been based on safety considerations (19). Naturalistic clinical data has found no meaningful differences in therapeutic outcomes with ITI ranging from 11 to 26 s (20), suggesting that treatment time could be reduced from the conventional 37.5 to 18 min without meaningful clinical differences. In the motor system, ITI ranging between 3 and 17 s produced inhibitory motor effects from successive single TMS pulses; however, a 1 s ITI, effectively becoming continuous 1 Hz stimulation, lost the suppressive effect (21). In contrast, ITIs of 4, 8, 16, and 32 s produced no difference in motor-evoked potentials of healthy humans using patterned 20 Hz rTMS (Figure 1D), although shorter ITI produced a marked disinhibition as measured by short intracortical inhibition (SICI) (22). The meaning of these different findings requires further exploration, but speculatively hints that different protocols may theoretically channel different neuronal populations with their corresponding symptoms or networks.

#### Pulse Number

Pulse number also appears to be consistent with the inverted U dose-response curve with further space for optimization (Figure 1A). Huang et al.'s (5) original theta burst findings that 600 pulses produced a more durable response than 300, but that doubling the iTBS pulse number to 1,200 actually produced inhibitory effects instead of the potentiating 600 pulses. More recent studies have produced similarly paradoxical findings that motor iTBS and cTBS at different pulse numbers produce differing facilitatory or inhibitory effects. Notably, Gamboa et al. (23) found that 1,200 iTBS pulses produced inhibitory motor effects, whereas McCalley et al. (24) reported that amongst 600, 1,200, 1,800, and 3,600 pulses of iTBS or cTBS, only 3,600 cTBS pulses produced excitatory motor effects. It is unclear whether these theta burst results in healthy adults over the motor cortex would translate clinically as iTBS is typically applied over multiple treatment sessions, at only 600 pulses per session, and over the prefrontal cortex.

An increasingly popular approach that can be utilized to study the effects of pulse number on brain response combines single pulses of TMS with electroencephalography (EEG) recordings with scalp electrodes, a method known as TMS-EEG (25). Since TMS-EEG directly measures the brain's response to TMS, researchers can assess the cortical effects of TMS outside of the motor system (e.g., in the prefrontal cortex) (26). Utilizing this approach, Desforges et al. (27) used TMS-EEG measured before and after 600, 1,200, or 1,800 pulses of iTBS over the left prefrontal cortex. The authors found that the number of pulses did not alter the cortical response, but that individual responses to different stimulation parameters varied widely. It is currently unclear how these prefrontal dose-response findings for pulse number might vary between single session studies compared with many sessions over a typical clinical course of TMS. However, there is preliminary evidence that a greater number of pulses could matter clinically. In an open-label trial, Cole et al. (28) showed that 1,800 pulses of iTBS elicited a remission rate of 90.5% (Figure 1F). However, due to this study altering other variables, such as session number and total number of sessions, it is difficult to draw definitive conclusions. While this study cannot conclusively tell us that the increased pulse number alone produced this strong antidepressant effect, it at least suggests that this higher pulse number does not appear to block clinical antidepressant efficacy.

Similarly, differing pulse number in conventional rTMS may also produce different effects. Che et al. (29) found that pulse number can cause divergent effects with 10 Hz rTMS, as 1,500 pulses, but not 3,000 pulses, produced analgesic effects. On the other hand, Fitzgerald et al. (30) tested the widely held clinical belief that more pulses per session is more effective, and found that 125 trains (5,625 pulses) vs. 50 (3,000 pulses) produced no differences in an randomized trial with 300 depressed patients. It is worth noting that pulse number has increased steadily from the earlier trials to today's clinical standard of 3,000 pulses (31), broadly suggesting momentum toward applying more pulses per session over time. As safety considerations also inform pulse number, it is important to note that Hadley et al. gave 6,800 pulses per session of open-label 10 Hz TMS to 19 depressed patients with good efficacy and no serious adverse events (32) (Figure 1E).

#### Session Number

TMS clinicians have anecdotally noticed that after a patient has plateaued in clinical improvement, continued treatment sessions could correspond with clinical worsening, again, consistent with the inverted U-shaped dose-response curve (Figure 1A). However, among non-responders from one clinical trial, 61% eventually remitted with ongoing twice weekly treatments for up to 16 weeks (33). These data suggest that the number of treatments may be titrated to individual response. One way to personalize session number might be through predictive modeling based on early response (or lack thereof) to rTMS treatment (34). Another intriguing approach used an adaptive algorithm to determine the number of sessions it would take to change the strength of resting state functional connectivity (rsFC) between a cortical parietal target and the hippocampus (35). Using this algorithm, Freedberg et al. (35) found that more than 4 sessions would be needed for 87.5% efficacy at changing rsFC connectivity in the hippocampal-cortical network. However, the exact number of sessions differed in each participant, again pointing to the potential utility of personalizing session number based on response. While repeated fMRI sessions to gauge or predict response could be cost prohibitive, EEG may provide an cheap and feasible alternative to establish desired network engagement, such as recently reported in the first TMS study to show changes in EEG microstates in TMS responders, but not non-responders (36). Researchers have also previously shown that the degree of iTBS-evoked EEG oscillations at baseline can predict iTBS-associated plasticity in the alpha and beta bands (37), providing a further use of EEG to predict rTMS response.

A parallel line of research has not only increased the overall number of sessions but also the number of sessions per day, known as “accelerated” TMS (aTMS). Interest in aTMS is based on two observations: good efficacy and rapid response, such as found in an early open label trial with 27 depressed patients (38). Unfortunately, not all studies agree and the rates of efficacy and response likely depend on the number of sessions per day, which have varied between 2 and 10 thus far. One randomized trial with 98 depression patients showed improved odds of remission with two sessions per day (39), while two other RCTs with 115 and 208 depressed patients showed no difference in remission or response rates, nor did they improve symptoms or speed of response (40, 41). While these trials included 2 or 3 sessions per day, Cole et al. (42) gave 29 depressed patients 10 daily sessions for 5 days, finding that active aTMS produced a 50% symptom reduction compared to just 11% for sham (Figure 1G). While this study has justifiably garnered wide attention, we cannot definitively state whether aTMS is solely responsible for this effect given multiple variables changed, including personalized rsFC targeting (see below).

#### Pulse Width

Altered pulse width may also have biologically meaningful effects. Peterchev et al. (43) varied pulse width between 30, 60, and 90 μs, finding that increased pulse width decreased the motor threshold (MT) by increasing pulse energy (Figure 1H). Casula et al. (44) not only found the same negative correlation between pulse width and MT, but also reported that wider pulse widths produced higher local EEG field potentials. In one study, varying pulse widths in 1 Hz rTMS produced divergent effects, pointing to the large impact that pulse widths can have; shorter pulse widths of 40 and 80 μs elicited canonical inhibitory 1 Hz effects while 120 μs pulse width 1 Hz was excitatory, possibly due to differential membrane properties of preferentially activated segment (45). Whether these findings reflect specificity of neuronal activation due to different pulse widths, or are simply a product of increased energy with wider pulse widths as suggested by findings from Shirota et al. (46), remains to be determined. While these findings are in healthy control subjects, perhaps next generation rTMS protocols will utilize wider pulse widths to improve efficacy, which may also produce less discomfort (47). Emerging engineering projects hold promise to make control over these variables more widely accessible (48).

#### Pulse Shape

Related to pulse width, pulse shape also clearly affects MEPs, but is perhaps the furthest from clinical adaptation (in large part due to most TMS machines not allowing the researcher to alter this parameter). Several principles emerge. First, biphasic (full sinusoidal) produces greater excitation than monophasic (half-sine) (49) and even two summated monophasic waveforms (50). However, pulse shape is more complicated since biphasic waves (widely used in clinical rTMS) stimulate neurons in both the posterior-anterior (PA) direction and then the anterior-posterior (AP) direction (Figure 1I). Each of these directions is thought to activate a distinct group of neurons. Therefore, the biphasic wave may be considered a summated activation of two neuronal populations; PA is activated first and provides the more robust excitatory effect, followed by a delayed and weaker AP activation (49). That different neuronal mechanisms may underlie low-frequency stimulation is suggested by the lack of effect on 1 Hz biphasic rTMS compared to robust inhibition with AP, PA, and rectangular pulse shapes (bidirectional pulse) (51). Taking this concept a step further, Jung et al. (52) applied quadri-pulse (q) TBS (666 Hz quadruplets with 1.5 ms interpulse intervals) and produced opposing motor plasticity effects when applied as single- or double-sine-waves, and as PA and AP directionality is applied. These interactions highlight the complexity of parameter interactions, and the importance of getting it right.

#### Frequency

rTMS frequency is perhaps the best studied parameter in depression trials, with common protocols including 20, 18, 10, and 5 Hz to the left DLPFC (53–57) as well as 1 Hz to the right DLPFC (Figure 1J) (58–60). However, recent evidence suggests that individualized frequency, matched with a patient's endogenous rhythm, may improve clinical outcome (61). Such personalized medicine is the focus of subsequent sections.

### Parameter Theme 2: Does Personalized Stimulation Target and Target Engagement Influence Treatment Response?

#### Functional Neuroimaging for Individualized Targeting

**Figure 2:**
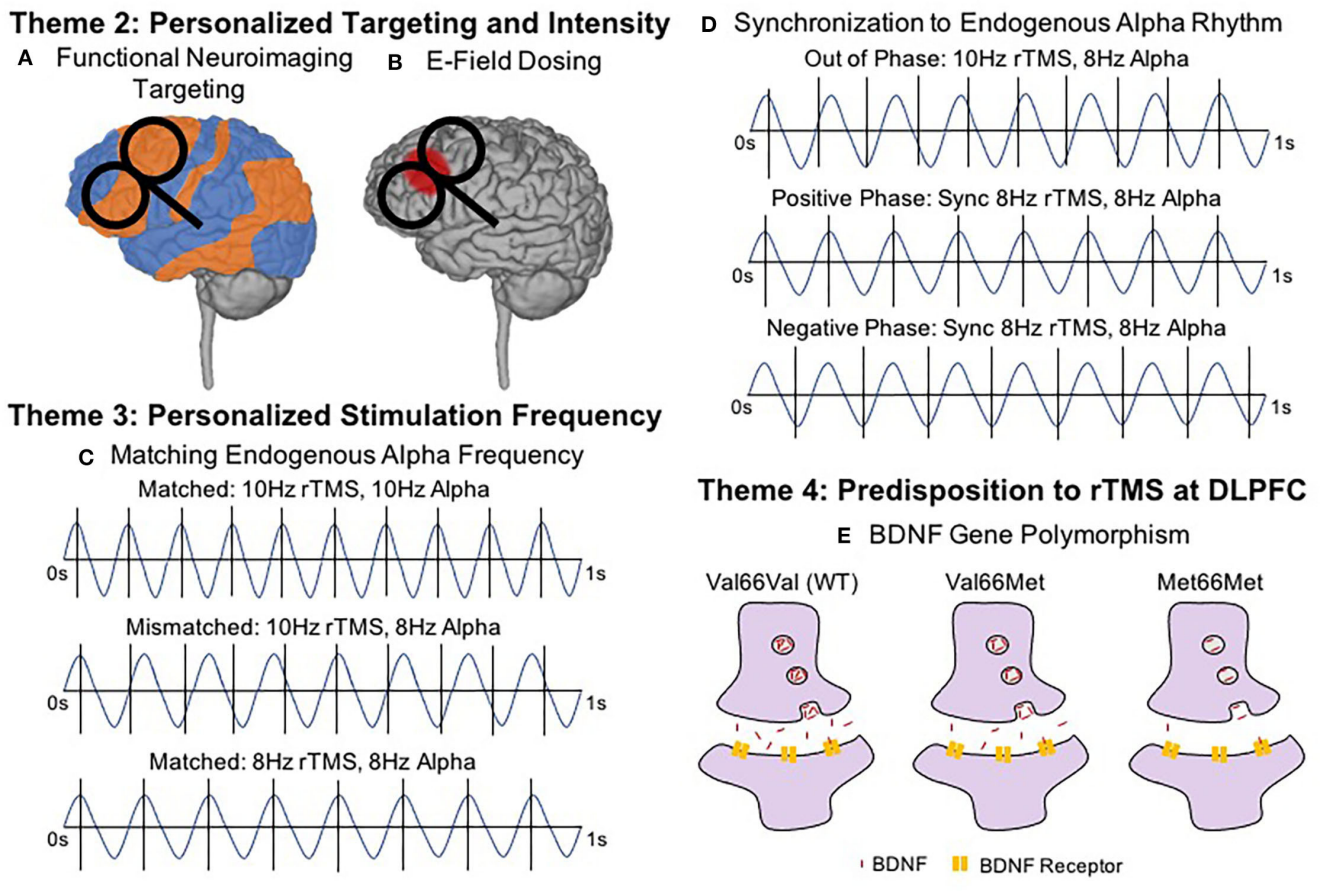
Key Brain States and Predispositions To Consider in Next Generation rTMS Therapies. **(A)** Functional Neuroimaging Targeting (Resting State Functional Connectivity; rsFC Targeting and concurrent TMS-fMRI). Target selection may become personalized based on functional connectivity and/or symptoms. Combining single pulses of TMS and measuring the blood oxygen level dependent (BOLD) signal may further help to individualize stimulation targets and possibly predict treatment course outcomes. **(B)** Electric Field (E-Field) Dosing. Intensity selection may utilize realistic head models and MRI-based E-field dosing to more precisely estimate the stimulation delivered to the target, particularly outside of the motor cortex. **(C)** Matching Endogenous Alpha Frequency. Patients with endogenous alpha rhythms closer to (or at) 10 Hz (top) responded better to 10 Hz rTMS than patients who were mismatched (middle). Most effective rTMS may involve stimulation at the endogenous frequency (bottom). **(D)** Synchronization to Endogenous Alpha Rhythm. Through closed-loop EEG, synchronized delivery of each rTMS train with an individualized endogenous alpha rhythm and aligning the timing of the TMS pulse with a specific phase of the waveform appears to further optimize rTMS effects. Here we show synchronized rTMS-EEG in three phases. *Out of Phase* describes when pulses are delivered without regard to endogenous oscillations (e.g., 10 Hz stimulation delivered for someone with endogenous 8 Hz oscillations). When rTMS is delivered *In Phase*, the pulses can be synchronized with the peak of each oscillation (i.e., *Positive Phase*) or at the trough of each oscillation (i.e., *Negative Phase*). Importantly, synchronizing the endogenous alpha rhythm could occur at any frequency, e.g., 8.5 Hz. **(E)** Predisposition to rTMS—BDNF Gene Polymorphism. Genetic predispositions may influence individual response to rTMS, such as the Val ??Met single nucleotide polymorphism found in the brain derived neurotrophic factor (BDNF) gene which impairs the normal plasticity response to rTMS protocols.

To date, the most common therapeutic target of rTMS for depression has been the left dorsolateral prefrontal cortex (DLPFC). However, the optimal target and method to identify that target within the left DLPFC remains an open discussion. Current standard clinical practice typically identifies the optimal prefrontal stimulation target using a set distance from the motor cortex (i.e., the 5 cm rule) or a probabilistic method of approximating the F3 EEG location (i.e., Beam F3). However, personalizing the rTMS target using resting state functional connectivity (rsFC) analyses may produce more clinically impactful results (Figure 2A). Weigand et al. (62) found that treatment response negatively correlated with rsFC strength between the DLPFC and subgenual anterior cingulate cortex (sgACC), two important nodes within the executive network. Several other studies have corroborated these findings (28, 63–66), and thus, it is possible that traditional targeting methods based on scalp measurements or EEG coordinates may be engaging the relevant networks only by chance and only at a group level [see comparisons of common targeting approaches in (64)]. In other words, using rsFC analyses to personalize stimulation target may be fruitful as each individual's optimal rsFC stimulation target often differs from the group averaged target location that may agree with the 5 cm or Beam F3 approaches. Moreover, standard targeting methods ignore the heterogeneity of depression, and emerging evidence supports the feasibility and importance of engaging depression subtypes and even symptoms (67). We can expect that what has been found with rTMS for depression could have relevance across brain disorders.

Another promising tool for identifying individualized rTMS targets involves combining single pulses of TMS and fMRI within the MR scanner environment, a technique called interleaved TMS-fMRI (68–70). By applying single pulses of TMS and recording the resulting blood oxygen level dependent (BOLD) signal, it is possible to directly and causally measure the brain's response to TMS (71). Notably, TMS-fMRI can record how single pulses of TMS affect brain activity, not only at the cortical surface, but also at distal regions of a brain network, such as the sgACC in depression (72, 73). Moreover, baseline TMS-fMRI response may be able to predict clinical outcome. In one study, depressed patients with more negative TMS-fMRI baseline responses in the sgACC corresponded with better symptom improvements (74). Thus, future research and clinical practice might utilize TMS-fMRI to determine optimal stimulation targets for rTMS treatment, or to predict the patients for whom rTMS may be most effective. Alternatively, less expensive and more accessible functional near-infrared spectroscopy (fNIRS), or diffuse optical tomography (DOT) could enable such targeting and even allow real-time visualization of the effects of varied rTMS protocols.

#### Stimulation Intensity

Even when the correct target is identified, it would produce no clinical benefits if the target were not adequately engaged by stimulation, such as with suboptimal stimulation intensity. In current practice, the stimulation intensity is derived from the motor threshold (MT), which relies on the assumption that cortical excitability in the motor cortex can accurately inform stimulation intensities at other cortical targets such as the prefrontal cortex. However, it remains unclear whether sufficient motor cortex activation equates to adequate prefrontal engagement, or how stimulating at a more optimized intensity might affect response rate. Historically, early TMS researchers in the 1990s proceeded with caution due to safety considerations, first applying rTMS at just 80% MT (75). Incrementally, these early researchers then incrementally increased rTMS intensities to 100% MT and eventually, the now widely adopted 120% MT based on evidence that greater scalp-to-cortex distance in older patients appeared to prevent high response to rTMS therapy at 100% MT intensities (76, 77), but that this could be overcome by individually adjusting for scalp-to-cortex distance (78).

A more recent tool is MRI-based electric field (E-field) modeling, which uses structural MRI-based tissue segmentation and varying tissue conductivities to more accurately estimate the amount of stimulation that reaches the cortex (79–81) and could be used to inform prospective dosing. Since E-field modeling is not dependent on the dubious assumption that motor cortical engagement can accurately estimate how much stimulation reaches prefrontal stimulation targets, E-field dosing could potentially inform higher fidelity, personalized stimulation intensities specifically for the prefrontal cortex or other rTMS targets. Thus, E-field dosing could prove particularly useful if the dose-response relationship between stimulation intensity and clinical response follows the inverted U-curve model with peak efficacy in the middle (Figure 1A). While largely untested, some extant dose-response experiments point to a stimulation intensity sweet spot that neither under- nor over-doses. Notably among these, Chung et al. (82) determined that 75% MT stimulation produced superior DLPFC TMS-evoked EEG potentials, rather than 50 or 100% MT. Similarly, Lee et al. (83) determined that subthreshold iTBS caused greater reductions in depressive symptoms than suprathreshold iTBS, again pointing to an optimal middle stimulation intensity. In retrospective E-field analyses of clinical rTMS for depression and smoking cessation, the prefrontal E-field magnitude from 120% MT stimulation did not linearly correlate with the percentage of symptom change (84, 85), possibly suggesting a non-linear dose-response relationship and perhaps peak efficacy with an optimized middle amount of stimulation. A remaining question is whether there is an optimal E-field dosing intensity, which itself could be prone to interindividual differences due to varied distributions of particular neuron types or different neurotransmitter concentrations between patients. To account for these potential individual differences, Caulfield et al. (86) have proposed to measure the E-field intensity at the MT to first determine an individual neuronal activation threshold by measuring a personalized MT and calculating the required stimulation intensity to replicate this motor E-field over the prefrontal stimulation target (Figure 2B). It remains to be seen whether optimized E-field dosing would improve clinical efficacy.

### Parameter Theme 3: How Does Endogenous Brain Activity or Brain State Affect RTMS Treatment Response?

#### Synchronization to Endogenous Brain Activity

Whereas conventional rTMS is applied with the same stimulation frequency across patients, emerging neuroimaging research could inform more personalized stimulation approaches. Leuchter et al. (61) systematically determined the resonant frequency of each subject by analyzing the effect of various rTMS stimulation frequencies (from 3 to 17 Hz) on electroencephalography (EEG)-based power and connectivity metrics. Intriguingly, those individuals with endogenous alpha rhythms closest to 10 Hz had the best treatment outcomes from standard 10 Hz rTMS for depression (87), hinting at the utility of using individualized stimulation frequencies (e.g., 8 Hz rTMS for someone with a strong inherent resonant frequency of 8 Hz) (Figure 2C). Similarly, Kundu et al. (88) found that the baseline beta band activity could predicted pulse-by-pulse variations in the TMS-evoked EEG response, again suggesting that endogenous brain activity impacts response to rTMS.

In a related but distinct effort, researchers have begun to study how rTMS pulses interact with brain rhythms in real time (i.e., synchronized TMS-EEG) (Figure 2D) (89). Research by Ferreri et al. (90, 91) retrospectively examined the relationship between ongoing EEG recordings and MEP amplitudes recorded concurrent with EEG, finding that there was greater EEG coupling on high MEP trials than low MEP trials. Keil et al. (92) also found that EEG activity impacts MEP response, as higher real-time beta-band EEG coherence with ongoing hand electromyographic (EMG) recordings produced stronger MEP amplitudes in a significant linear relationship. Putting these concepts from single pulse TMS studies together, researchers have begun to test the effects of real-time, closed-loop rTMS-EEG synchronization and whether this causes meaningful neural or behavioral changes compared to unsynchronized rTMS-EEG. These cutting edge synchronized rTMS-EEG experiments have found that personalizing and synchronizing rTMS and iTBS pulse timing to endogenous EEG rhythms in the brain circuit of interest can significantly increase prefrontal EEG response (93) and MEP amplitudes (94) in comparison to unsynchronized conditions.

Increasingly nuanced approaches also consider the importance of EEG phase and whether the rTMS pulse is delivered at the peak (positive phase) or trough (negative phase) of brain rhythms (Figure 2D). In particular, Momi et al. (95) have found that phase-locking rTMS pulses to the negative phase of the pulse elicits stronger mu synchrony throughout the sensorimotor network when compared to synchronizing pulses to the positive phase of the EEG signal. In the first application of these synchronized rTMS-EEG approaches in a clinical population, Zrenner et al. (96) demonstrated the feasibility and utility of synchronizing iTBS with alpha oscillations in the prefrontal cortex of MDD patients. These researchers found that alpha-synchronized iTBS caused significantly larger decreases in resting state alpha activity at the left prefrontal target, suggesting that synchronized rTMS-EEG could produce meaningful clinical results if applied over an entire treatment course (96). An ongoing clinical trial (NCT03421808) is attempting to address the therapeutic effects of synchronizing rTMS-EEG for depression over a treatment course.

Lastly, we would be remiss if we did not discuss a prior large scale attempt to synchronize TMS with endogenous alpha rhythm for depression using a technology known as low field synchronized TMS (sTMS) (97). Low field sTMS applies weak magnetic fields using midline rotating magnets that can match the personalized, EEG-determined oscillatory frequency for each depression patient. While the antidepressant effects of sTMS were initially promising (97), the pivotal trial showed no significant differences between active and sham sTMS (98). However, it is important to note that the mechanism of low field sTMS is fundamentally different than patterned rTMS or iTBS, with the maximum magnetic field change over time in low field sTMS ~1000x lower than conventional rTMS (97). Thus, this emerging concept of matching or synchronizing rTMS or iTBS with endogenous brain oscillations remains a promising area of research.

#### Brain State

Consistent with principles from fundamental LTP studies, the state of the brain at the time of stimulation may affect treatment outcome. Isserles et al. (99) have demonstrated what we have long assumed, that it matters what our brain is doing during rTMS. They found that reading a script that promoting positive cognitive-emotional activation leads to greater antidepressant effects than does negative or neutral scripts. A further method of priming the brain for rTMS could be concurrent aerobic exercise, which review articles have proposed could complement the therapeutic effects or rTMS due to aerobic exercise priming synaptic plasticity (100, 101). Surprisingly, these approaches remain untested in large scale clinical trials. Thus, along with personalized cognitive engagement, next generation rTMS may include capitalizing on brain state at the macroscale (i.e., cognitive engagement) and microscale levels (i.e., synchronized with phase of endogenous waveforms).

### Parameter Theme 4: Are Some Brains Naturally Receptive vs. Resistant to RTMS?

#### rsFC States and Genetic Predispositions

Lastly, inherent characteristics may portend individual response to rTMS. In addition to individual baseline differences in rsFC predicting degree of antidepressant rTMS effect, some researchers have identified predispositions that portend the likelihood of rTMS response. Notably, Drysdale et al. (102) identified four distinct rsFC states that relate to different symptom clusters (i.e., dysphoric or anxiosomatic), and found that more anxious patients responded preferentially to dorsomedial (DM) PFC rTMS compared to predominantly dysphoric patients by nearly 4-fold. Perhaps baseline rsFC analyses could predict ideal candidates for rTMS at a given target, with non-ideal candidates provided with alternative therapeutic options.

Genetic predispositions can also influence rTMS response. Cheeran et al. (103) characterized how the heterozygous Val66Met polymorphism, which is associated with lower concentration of brain derived neurotrophic factor (BDNF), has been associated with decreased rTMS plasticity over the motor cortex compared to homozygous Val66Val individuals (103) (Figure 2E). Subsequent research has confirmed this seminal finding with the Val66Val genotype associated with the highest TMS motor evoked response (104, 105), Met66Met polymorphism associated with the lowest TMS motor evoked response (106), and BDNF gene predicting up to 59% of between-subject variability of MEP responses (107). These findings in healthy adults over the motor system also hold clinical validity, as the Val66Val genotype is most likely to respond positively to rTMS in stroke (108). Just as genetics are gaining traction as a predictor of pharmacologic response, we may find a useful guide to stimulation type and parameters in our genotypes. For instance, researchers have found that increasing the number of days of motor training can overcome the natural predisposition for Val to Met polymorphism to cause lower cortical responses (109); in a similar vein, perhaps an increased number of rTMS pulses or sessions could overcome individual genetic predilections to respond/not respond to brain stimulation treatments.

## Discussion

In this mini-review, we outlined four parameter themes guiding the next generation of rTMS treatments. Implicit in many of these studies is that cortical plasticity (i.e., MEPs) may provide a surrogate for clinical response. Indeed, motor cortex plasticity assessed by MEP response to a 10 Hz protocol reliably predicted whether depressed patients respond to rTMS (18). We envision future rTMS will be delivered to rsFC-determined targets at intensities determined by energy delivered to the cortex, using optimized pulse number, train duration, intertrain intervals, and pulse widths/shapes, with frequency personalized to endogenous alpha-rhythms and even synchronized to coincide with the timing and phase of the endogenous waveforms. Future research is needed to define the “curve” of each parameter on plasticity and clinical response at the group level, to determine how these parameters interact, and to ultimately personalize these parameters. A tiered approach may prove most practical considering the cost-benefit ratio of these complex fMRI and EEG-based techniques, with more advanced and expensive techniques reserved for those not remitting with traditional methods.

## Author Contributions

KC and JB conceived of the review, wrote the first draft, created the figures, and edited the manuscript. Both authors contributed to the article and approved the submitted version.

## Funding

This work was supported by an US NIH/NINDS grant (1F31NS126019) to KC, and US NIH/NIGMS grant (P20GM130452) Center for Biomedical Research Excellence, Center for Neuromodulation to JB.

## Conflict of Interest

The authors declare that the research was conducted in the absence of any commercial or financial relationships that could be construed as a potential conflict of interest.

## Publisher's Note

All claims expressed in this article are solely those of the authors and do not necessarily represent those of their affiliated organizations, or those of the publisher, the editors and the reviewers. Any product that may be evaluated in this article, or claim that may be made by its manufacturer, is not guaranteed or endorsed by the publisher.

## References

[1] O'Reardon JP Solvason HB Janicak PG Sampson S Isenberg KE Nahas Z. Efficacy and safety of transcranial magnetic stimulation in the acute treatment of major depression: a multisite randomized controlled trial. Biol Psychiatry. (2007) 62:1208–16. 10.1016/j.biopsych.2007.01.018 17573044

[2] Carmi L Tendler A Bystritsky A Hollander E Blumberger DM Daskalakis J. Efficacy and safety of deep transcranial magnetic stimulation for obsessive-compulsive disorder: a prospective multicenter randomized double-blind placebo-controlled trial. Am J Psychiatry. (2019) 176:931–8. 10.1176/appi.ajp.2019.18101180 31109199

[3] Zangen A Moshe H Martinez D Barnea-Ygael N Vapnik T Bystritsky A. Repetitive transcranial magnetic stimulation for smoking cessation: a pivotal multicenter double-blind randomized controlled trial. World Psychiatry. (2021) 20:397–404. 10.1002/wps.20905 34505368 PMC8429333

[4] Carpenter LL Janicak PG Aaronson ST Boyadjis T Brock DG Cook IA. Transcranial magnetic stimulation (TMS) for major depression: a multisite, naturalistic, observational study of acute treatment outcomes in clinical practice. Depress Anxiety. (2012) 29:587–96. 10.1002/da.21969 22689344

[5] Huang YZ Edwards MJ Rounis E Bhatia KP Rothwell JC. Theta burst stimulation of the human motor cortex. Neuron. (2005) 45:201–6. 10.1016/j.neuron.2004.12.033 15664172

[6] Di Lazzaro V Dileone M Pilato F Capone F Musumeci G Ranieri F. Modulation of motor cortex neuronal networks by rTMS: comparison of local and remote effects of six different protocols of stimulation. J Neurophysiol. (2011) 105:2150–6. 10.1152/jn.00781.2010 21346213

[7] Zhao S Kong J Li S Tong Z Yang C Zhong H. Randomized controlled trial of four protocols of repetitive transcranial magnetic stimulation for treating the negative symptoms of schizophrenia. Shanghai Arch Psychiatry. (2014) 26:15–21. 10.3969/j.issn.1002-0829.2014.01.003 25114477 PMC4117998

[8] Blumberger DM Vila-Rodriguez F Thorpe KE Feffer K Noda Y Giacobbe P. Effectiveness of theta burst versus high-frequency repetitive transcranial magnetic stimulation in patients with depression (THREE-D): a randomised non-inferiority trial. Lancet. (2018) 391:1683–92. 10.1016/S0140-6736(18)30295-2 29726344

[9] Tsai PY Lin WS Tsai KT Kuo CY Lin PH. High-frequency versus theta burst transcranial magnetic stimulation for the treatment of poststroke cognitive impairment in humans. J Psychiatry Neurosci. (2020) 45:262–70. 10.1503/jpn.190060 32159313 PMC7828923

[10] Philip NS Barredo J Aiken E Larson V Jones RN Shea MT. Theta-burst transcranial magnetic stimulation for posttraumatic stress disorder. Am J Psychiatry. (2019) 176:939–48. 10.1176/appi.ajp.2019.18101160 31230462 PMC6824981

[11] Petrosino NJ Wout-Frank MV Aiken E Swearingen HR Barredo J Zandvakili A. One-year clinical outcomes following theta burst stimulation for post-traumatic stress disorder. Neuropsychopharmacology. (2020) 45:940–6. 10.1038/s41386-019-0584-4 31794974 PMC7162862

[12] Philip NS Doherty RA Faucher C Aiken E van ‘t Wout-Frank M. Transcranial magnetic stimulation for posttraumatic stress disorder and major depression: comparing commonly used clinical protocols. J Traumatic Stress. (2021) 35:101–8. 10.1002/jts.22686 33973681 PMC8581062

[13] Teo JT Swayne OB Rothwell JC. Further evidence for NMDA-dependence of the after-effects of human theta burst stimulation. Clin Neurophysiol. (2007) 118:1649–51. 10.1016/j.clinph.2007.04.010 17502166

[14] Selby B MacMaster FP Kirton A McGirr A. d-cycloserine blunts motor cortex facilitation after intermittent theta burst transcranial magnetic stimulation: a double-blind randomized placebo-controlled crossover study. Brain Stimul. (2019) 12:1063–5. 10.1016/j.brs.2019.03.026 30914260

[15] Brown JC DeVries WH Korte JE Sahlem GL Bonilha L Short EB. NMDA receptor partial agonist, d-cycloserine, enhances 10 Hz rTMS-induced motor plasticity, suggesting long-term potentiation (LTP) as underlying mechanism. Brain Stimul. (2020) 13:530–2. 10.1016/j.brs.2020.01.005 32289670 PMC7224691

[16] Brown JC Yuan S DeVries WH Armstrong NM Korte JE Sahlem GL. NMDA-receptor agonist reveals LTP-like properties of 10-Hz rTMS in the human motor cortex. Brain Stimul. (2021) 14:619–21. 10.1016/j.brs.2021.03.016 33794339 PMC8164996

[17] Jung SH Shin JE Jeong YS Shin HI. Changes in motor cortical excitability induced by high-frequency repetitive transcranial magnetic stimulation of different stimulation durations. Clin Neurophysiol. (2008) 119:71–9. 10.1016/j.clinph.2007.09.124 18039593

[18] Oliveira-Maia AJ Press D Pascual-Leone A. Modulation of motor cortex excitability predicts antidepressant response to prefrontal cortex repetitive transcranial magnetic stimulation. Brain Stimul. (2017) 10:787–94. 10.1016/j.brs.2017.03.013 28438543 PMC5576557

[19] Chen R Gerloff C Classen J Wassermann EM Hallett M Cohen LG. Safety of different inter-train intervals for repetitive transcranial magnetic stimulation and recommendations for safe ranges of stimulation parameters. Electroencephalogr Clin Neurophysiol. (1997) 105:415–21. 10.1016/S0924-980X(97)00036-2 9448642

[20] Carpenter L Aaronson S Hutton TM Mina M Pages K Verdoliva S. Comparison of clinical outcomes with two Transcranial Magnetic Stimulation treatment protocols for major depressive disorder. Brain Stimul. (2021) 14:173–80. 10.1016/j.brs.2020.12.003 33346068

[21] Pitkänen M Kallioniemi E Julkunen P. Effect of inter-train interval on the induction of repetition suppression of motor-evoked potentials using transcranial magnetic stimulation. PLoS ONE. (2017) 12:e0181663. 10.1371/journal.pone.0181663 28723977 PMC5517025

[22] Cash RFH Dar A Hui J De Ruiter L Baarbé J Fettes P. Influence of inter-train interval on the plastic effects of rTMS. Brain Stimul. (2017) 10:630–6. 10.1016/j.brs.2017.02.012 28285889

[23] Gamboa OL Antal A Moliadze V Paulus W. Simply longer is not better: reversal of theta burst after-effect with prolonged stimulation. Exp Brain Res. (2010) 204:181–7. 10.1007/s00221-010-2293-4 20567808 PMC2892066

[24] McCalley DM Lench DH Doolittle JD Imperatore JP Hoffman M Hanlon CA. Determining the optimal pulse number for theta burst induced change in cortical excitability. Sci Rep. (2021) 11:8726. 10.1038/s41598-021-87916-2 33888752 PMC8062542

[25] Taylor PCJ Walsh V Eimer M. Combining TMS and EEG to study cognitive function and cortico-cortico interactions. Behav Brain Res. (2008) 191:141–7. 10.1016/j.bbr.2008.03.033 18485496 PMC2779459

[26] Tremblay S Rogasch NC Premoli I Blumberger DM Casarotto S Chen R. Clinical utility and prospective of TMS–EEG. Clin Neurophysiol. (2019) 130:802–44. 10.1016/j.clinph.2019.01.001 30772238

[27] Desforges M Hadas I Mihov B Morin Y Rochette Braün M Lioumis P. Dose-response of intermittent theta burst stimulation of the prefrontal cortex: a TMS-EEG study. Clin Neurophysiol. (2022) 136:158–72. 10.1016/j.clinph.2021.12.018 35183861

[28] Cole EJ Stimpson KH Bentzley BS Gulser M Cherian K Tischler C. Stanford accelerated intelligent neuromodulation therapy for treatment-resistant depression. Am J Psychiatry. (2020) 177:716–26. 10.1176/appi.ajp.2019.19070720 32252538

[29] Che X Fitzgibbon BM Ye Y Wang J Luo H Fitzgerald PB. Characterising the optimal pulse number and frequency for inducing analgesic effects with motor cortex rTMS. Brain Stim. (2021) 14:1081–3. 10.1016/j.brs.2021.06.015 34224868

[30] Fitzgerald PB Hoy KE Reynolds J Singh A Gunewardene R Slack C. A pragmatic randomized controlled trial exploring the relationship between pulse number and response to repetitive transcranial magnetic stimulation treatment in depression. Brain Stim. (2020) 13:145–52. 10.1016/j.brs.2019.09.001 31521543

[31] George MS Lisanby SH Avery D McDonald WM Durkalski V Pavlicova M. Daily left prefrontal transcranial magnetic stimulation therapy for major depressive disorder: a sham-controlled randomized trial. Arch Gen Psychiatry. (2010) 67:507–16. 10.1001/archgenpsychiatry.2010.46 20439832

[32] Hadley D Anderson BS Borckardt JJ Arana A Li X Nahas Z. Safety, tolerability, and effectiveness of high doses of adjunctive daily left prefrontal repetitive transcranial magnetic stimulation for treatment-resistant depression in a clinical setting. J ect. (2011) 27:18–25. 10.1097/YCT.0b013e3181ce1a8c 21343710

[33] Yip AG George MS Tendler A Roth Y Zangen A Carpenter LL. 61% of unmedicated treatment resistant depression patients who did not respond to acute TMS treatment responded after four weeks of twice weekly deep TMS in the Brainsway pivotal trial. Brain Stimul. (2017) 10:847–9. 10.1016/j.brs.2017.02.013 28330592

[34] Berlow YA Zandvakili A Philip NS. The clinical utility of imaging-defined biotypes of depression and transcranial magnetic stimulation: a decision curve analysis. Brain Stim. (2020) 13:1069–70. 10.1016/j.brs.2020.04.016 32360391 PMC7367150

[35] Freedberg M Reeves JA Toader AC Hermiller MS Kim E Haubenberger D. Optimizing hippocampal-cortical network modulation via repetitive transcranial magnetic stimulation: a dose-finding study using the continual reassessment method. Neuromodulation. (2020) 23:366–72. 10.1111/ner.13052 31667947 PMC7657658

[36] Gold MC Yuan S Tirrell E Kronenberg EF Kang JWD Hindley L. Large-scale EEG neural network changes in response to therapeutic TMS. Brain Stimul. (2022) 15:316–25. 10.1016/j.brs.2022.01.007 35051642 PMC8957581

[37] Leodori G Fabbrini A De Bartolo MI Costanzo M Asci F Palma V. Cortical mechanisms underlying variability in intermittent theta-burst stimulation-induced plasticity: a TMS-EEG study. Clin Neurophysiol. (2021) 132:2519–31. 10.1016/j.clinph.2021.06.021 34454281

[38] McGirr A Van den Eynde F Tovar-Perdomo S Fleck MP Berlim MT. Effectiveness and acceptability of accelerated repetitive transcranial magnetic stimulation (rTMS) for treatment-resistant major depressive disorder: an open label trial. J Affective Disord. (2015) 173:216–20. 10.1016/j.jad.2014.10.068 25462419

[39] Theleritis C Sakkas P Paparrigopoulos T Vitoratou S Tzavara C Bonaccorso S. Two versus one high-frequency repetitive transcranial magnetic stimulation session per day for treatment-resistant depression: a randomized sham-controlled trial. J ect. (2017) 33:190–7. 10.1097/YCT.0000000000000387 28072660

[40] Fitzgerald PB Hoy KE Elliot D McQueen RS Wambeek LE Daskalakis ZJ. Accelerated repetitive transcranial magnetic stimulation in the treatment of depression. Neuropsychopharmacology. (2018) 43:1565–72. 10.1038/s41386-018-0009-9 29467437 PMC5983543

[41] Blumberger DM Vila-Rodriguez F Wang W Knyahnytska Y Butterfield M Noda Y. A randomized sham controlled comparison of once vs twice-daily intermittent theta burst stimulation in depression: a Canadian rTMS treatment and biomarker network in depression (CARTBIND) study. Brain Stim. (2021) 14:1447–55. 10.1016/j.brs.2021.09.003 34560319

[42] Cole EJ Phillips AL Bentzley BS Stimpson KH Nejad R Barmak F. Stanford Neuromodulation Therapy (SNT): a double-blind randomized controlled trial. Am J Psychiatry. (2021) 179:132–41. 10.1176/appi.ajp.2021.20101429 34711062

[43] Peterchev AV Goetz SM Westin GG Luber B Lisanby SH. Pulse width dependence of motor threshold and input–output curve characterized with controllable pulse parameter transcranial magnetic stimulation. Clin Neurophysiol. (2013) 124:1364–72. 10.1016/j.clinph.2013.01.011 23434439 PMC3664250

[44] Casula EP Rocchi L Hannah R Rothwell JC. Effects of pulse width, waveform and current direction in the cortex: a combined cTMS-EEG study. Brain Stimul. (2018) 11:1063–70. 10.1016/j.brs.2018.04.015 29709505

[45] Halawa I Shirota Y Neef A Sommer M Paulus W. Neuronal tuning: selective targeting of neuronal populations via manipulation of pulse width and directionality. Brain Stimul. (2019) 12:1244–52. 10.1016/j.brs.2019.04.012 31085123

[46] Shirota Y Sommer M Paulus W. Strength-duration relationship in paired-pulse Transcranial Magnetic Stimulation (TMS) and its implications for repetitive TMS. Brain Stimul. (2016) 9:755–61. 10.1016/j.brs.2016.04.019 27234142

[47] Peterchev AV Luber B Westin GG Lisanby SH. Pulse width affects scalp sensation of transcranial magnetic stimulation. Brain Stimul. (2017) 10:99–105. 10.1016/j.brs.2016.09.007 28029593 PMC5241181

[48] Zeng Z Koponen LM Hamdan R Li Z Goetz SM Peterchev AV. Modular multilevel TMS device with wide output range and ultrabrief pulse capability for sound reduction. J Neural Eng. (2022) 19:026008. 10.1088/1741-2552/ac572c 35189604 PMC9425059

[49] Sommer M Alfaro A Rummel M Speck S Lang N Tings T. Half sine, monophasic and biphasic transcranial magnetic stimulation of the human motor cortex. Clin Neurophysiol. (2006) 117:838–44. 10.1016/j.clinph.2005.10.029 16495145

[50] Delvendahl I Gattinger N Berger T Gleich B Siebner HR Mall V. The role of pulse shape in motor cortex transcranial magnetic stimulation using full-sine stimuli. PLoS ONE. (2014) 9:e115247. 10.1371/journal.pone.0115247 25514673 PMC4267841

[51] Goetz SM Luber B Lisanby SH Murphy DLK Kozyrkov IC Grill WM. Enhancement of neuromodulation with novel pulse shapes generated by controllable pulse parameter transcranial magnetic stimulation. Brain Stim. (2016) 9:39–47. 10.1016/j.brs.2015.08.013 26460199 PMC5517314

[52] Jung NH Gleich B Gattinger N Kalb A Fritsch J Asenbauer E. Double-sine-wave quadri-pulse theta burst stimulation of precentral motor hand representation induces bidirectional changes in corticomotor excitability. Front Neurol. (2021) 12:673560. 10.3389/fneur.2021.673560 34262522 PMC8273174

[53] Levkovitz Y Isserles M Padberg F Lisanby SH Bystritsky A Xia G. Efficacy and safety of deep transcranial magnetic stimulation for major depression: a prospective multicenter randomized controlled trial. World Psychiatry. (2015) 14:64–73. 10.1002/wps.20199 25655160 PMC4329899

[54] Philip NS Ridout SJ Albright SE Sanchez G Carpenter LL. 5-Hz transcranial magnetic stimulation for comorbid posttraumatic stress disorder and major depression. J Trauma Stress. (2016) 29:93–6. 10.1002/jts.22065 26748883 PMC4849266

[55] George MS Nahas Z Molloy M Speer AM Oliver NC Li XB. A controlled trial of daily left prefrontal cortex TMS for treating depression. Biol Psychiatry. (2000) 48:962–70. 10.1016/S0006-3223(00)01048-9 11082469

[56] Berlim MT van den Eynde F Tovar-Perdomo S Daskalakis ZJ. Response, remission and drop-out rates following high-frequency repetitive transcranial magnetic stimulation (rTMS) for treating major depression: a systematic review and meta-analysis of randomized, double-blind and sham-controlled trials. Psychol Med. (2014) 44:225–39. 10.1017/S0033291713000512 23507264

[57] Sehatzadeh S Daskalakis ZJ Yap B Tu H-A Palimaka S Bowen JM. Unilateral and bilateral repetitive transcranial magnetic stimulation for treatment-resistant depression: a meta-analysis of randomized controlled trials over 2 decades. J Psychiatry Neurosci. (2019) 44:151–63. 10.1503/jpn.180056 30720259 PMC6488490

[58] Berlim MT Van den Eynde F Jeff Daskalakis Z. Clinically meaningful efficacy and acceptability of low-frequency repetitive transcranial magnetic stimulation (rTMS) for treating primary major depression: a meta-analysis of randomized, double-blind and sham-controlled trials. Neuropsychopharmacology. (2013) 38:543–51. 10.1038/npp.2012.237 23249815 PMC3572468

[59] Fitzgerald PB Hoy K Daskalakis ZJ Kulkarni J. A randomized trial of the anti-depressant effects of low- and high-frequency transcranial magnetic stimulation in treatment-resistant depression. Depress Anxiety. (2009) 26:229–34. 10.1002/da.20454 19105212

[60] Chen J Zhou C Wu B Wang Y Li Q Wei Y. Left versus right repetitive transcranial magnetic stimulation in treating major depression: a meta-analysis of randomised controlled trials. Psychiatry Res. (2013) 210:1260–4. 10.1016/j.psychres.2013.09.007 24113125

[61] Leuchter AF Wilson AC Vince-Cruz N Corlier J. Novel method for identification of individualized resonant frequencies for treatment of Major Depressive Disorder (MDD) using repetitive Transcranial Magnetic Stimulation (rTMS): a proof-of-concept study. Brain Stimul. (2021) 14:1373–83. 10.1016/j.brs.2021.08.011 34425244

[62] Weigand A Horn A Caballero R Cooke D Stern AP Taylor SF. Prospective validation that subgenual connectivity predicts antidepressant efficacy of transcranial magnetic stimulation sites. Biol Psychiatry. (2018) 84:28–37. 10.1016/j.biopsych.2017.10.028 29274805 PMC6091227

[63] Fox MD Buckner RL White MP Greicius MD Pascual-Leone A. Efficacy of transcranial magnetic stimulation targets for depression is related to intrinsic functional connectivity with the subgenual cingulate. Biol Psychiatry. (2012) 72:595–603. 10.1016/j.biopsych.2012.04.028 22658708 PMC4120275

[64] Cash RFH Weigand A Zalesky A Siddiqi SH Downar J Fitzgerald PB. Using brain imaging to improve spatial targeting of transcranial magnetic stimulation for depression. Biol Psychiatry. (2021) 90:689–700. 10.1016/j.biopsych.2020.05.033 32800379

[65] Cash RFH Cocchi L Lv J Fitzgerald PB Zalesky A. Functional magnetic resonance imaging-guided personalization of transcranial magnetic stimulation treatment for depression. JAMA Psychiatry. (2021) 78:337–9. 10.1001/jamapsychiatry.2020.3794 33237320 PMC7689561

[66] Ge R Downar J Blumberger DM Daskalakis ZJ Vila-Rodriguez F. Functional connectivity of the anterior cingulate cortex predicts treatment outcome for rTMS in treatment-resistant depression at 3-month follow-up. Brain Stim. (2020) 13:206–14. 10.1016/j.brs.2019.10.012 31668646

[67] Siddiqi SH Taylor SF Cooke D Pascual-Leone A George MS Fox MD. Distinct symptom-specific treatment targets for circuit-based neuromodulation. Am J Psychiatry. (2020) 177:435–46. 10.1176/appi.ajp.2019.19090915 32160765 PMC8396109

[68] Bestmann S Ruff CC Blankenburg F Weiskopf N Driver J Rothwell JC. Mapping causal interregional influences with concurrent TMS–fMRI. Exp Brain Res. (2008) 191:383. 10.1007/s00221-008-1601-8 18936922

[69] Bergmann TO Varatheeswaran R Hanlon CA Madsen KH Thielscher A Siebner HR. Concurrent TMS-fMRI for causal network perturbation and proof of target engagement. Neuroimage. (2021) 237:118093. 10.1016/j.neuroimage.2021.118093 33940146

[70] Bohning D Shastri A McConnell K Nahas Z Lorberbaum J Roberts D. A combined TMS/fMRI study of intensity-dependent TMS over motor cortex. Biol Psychiatry. (1999) 45:385–94. 10.1016/S0006-3223(98)00368-0 10071706

[71] Peters JC Reithler J Schuhmann T de Graaf T Uludag K Goebel R. On the feasibility of concurrent human TMS-EEG-fMRI measurements. J Neurophysiol. (2012) 109:1214–27. 10.1152/jn.00071.2012 23221407 PMC3569123

[72] Vink JJT Mandija S Petrov PI van den Berg CAT Sommer IEC Neggers SFW. A novel concurrent TMS-fMRI method to reveal propagation patterns of prefrontal magnetic brain stimulation. Human Brain Mapp. (2018) 39:4580–92. 10.1002/hbm.24307 30156743 PMC6221049

[73] Tik M Woletz M Schuler A-L Vasileiadi M Cash R Zalesky A. Concurrent TMS/fMRI validates MDD target network engagement. Brain Stim. (2021) 14:1710. 10.1016/j.brs.2021.10.401

[74] Oathes D. Depression improvement from rTMS facilitated by subgenual cingulate engagement indexed by interleaved TMS/fMRI. Biol Psychiatry. (2021) 89:S261–2. 10.1016/j.biopsych.2021.02.654

[75] George MS Wassermann EM Williams WA Callahan A Ketter TA Basser P. Daily repetitive transcranial magnetic stimulation (rTMS) improves mood in depression. Neuroreport. (1995) 6:1853–6. 10.1097/00001756-199510020-00008 8547583

[76] Figiel GS Epstein C McDonald WM Amazon-Leece J Figiel L Saldivia A. The use of rapid-Rate Transcranial Magnetic Stimulation (rTMS) in refractory depressed patients. J Neuropsychiatry Clin Neurosci. (1998) 10:20–5. 10.1176/jnp.10.1.20 9547462

[77] Kozel FA Nahas Z deBrux C Molloy M Lorberbaum JP Bohning D. How coil-cortex distance relates to age, motor threshold, and antidepressant response to repetitive transcranial magnetic stimulation. J Neuropsychiatry Clin Neurosci. (2000) 12:376–84. 10.1176/jnp.12.3.376 10956572

[78] Nahas Z Li X Kozel FA Mirzki D Memon M Miller K. Safety and benefits of distance-adjusted prefrontal transcranial magnetic stimulation in depressed patients 55-75 years of age: a pilot study. Depress Anxiety. (2004) 19:249–56. 10.1002/da.20015 15274174

[79] Saturnino GB Puonti O Nielsen JD Antonenko D Madsen KH Thielscher A. SimNIBS 2.1: a comprehensive pipeline for individualized electric field modelling for transcranial brain stimulation. In: Makarov S, Horner M, Noetscher G, editors. Brain and Hum Bod Model. Cham: Springer Copyright 2019 (2019). p. 3–25. 31725247

[80] Huang Y Liu AA Lafon B Friedman D Dayan M Wang X. Measurements and models of electric fields in the in vivo human brain during transcranial electric stimulation. eLife. (2017) 6:e18834. 10.7554/eLife.18834 28169833 PMC5370189

[81] Van Hoornweder S Meesen R Caulfield KA. On the importance of using both T1-weighted and T2-weighted structural magnetic resonance imaging scans to model electric fields induced by non-invasive brain stimulation in SimNIBS. Brain Stimul. (2022) 15:641–44. 10.1016/j.brs.2022.04.010 35436593

[82] Chung SW Rogasch NC Hoy KE Sullivan CM Cash RF Fitzgerald PB. Impact of different intensities of intermittent theta burst stimulation on the cortical properties during TMS-EEG and working memory performance. Human Brain Mapp. (2018) 39:783–802. 10.1002/hbm.23882 29124791 PMC6866298

[83] Lee JC Corlier J Wilson AC Tadayonnejad R Marder KG Ngo D. Subthreshold stimulation intensity is associated with greater clinical efficacy of intermittent theta-burst stimulation priming for Major Depressive Disorder. Brain Stim. (2021) 14:1015–21. 10.1016/j.brs.2021.06.008 34186465

[84] Caulfield KA Li X George MS. A reexamination of motor and prefrontal TMS in tobacco use disorder: time for personalized dosing based on electric field modeling? Clin Neurophysiol. (2021) 132:2199–207. 10.1016/j.clinph.2021.06.015 34298414 PMC8384673

[85] Deng Z-D Liston C Gunning FM Dubin MJ Fridgeirsson EA Lilien J. Electric field modeling for transcranial magnetic stimulation and electroconvulsive therapy. In: Makarov S, Horner M, Noetscher G, editors. Brain and Human Body Modeling. Cham: Springer (2019) 75–84. 10.1007/978-3-030-21293-3_4 31725245

[86] Caulfield KA Li X George MS. Four electric field modeling methods of Dosing Prefrontal Transcranial Magnetic Stimulation (TMS): introducing APEX MT dosimetry. Brain Stimul. (2021) 14:1032–4. 10.1016/j.brs.2021.06.012 34186248 PMC8866033

[87] Corlier J Carpenter LL Wilson AC Tirrell E Gobin AP Kavanaugh B. The relationship between individual alpha peak frequency and clinical outcome with repetitive Transcranial Magnetic Stimulation (rTMS) treatment of Major Depressive Disorder (MDD). Brain Stimul. (2019) 12:1572–8. 10.1016/j.brs.2019.07.018 31378603

[88] Kundu B Johnson JS Postle BR. Prestimulation phase predicts the TMS-evoked response. J Neurophysiol. (2014) 112:1885–93. 10.1152/jn.00390.2013 25008413 PMC4200008

[89] Zrenner C Desideri D Belardinelli P Ziemann U. Real-time EEG-defined excitability states determine efficacy of TMS-induced plasticity in human motor cortex. Brain Stimul. (2018) 11:374–89. 10.1016/j.brs.2017.11.016 29191438

[90] Ferreri F Vecchio F Ponzo D Pasqualetti P Rossini PM. Time-varying coupling of EEG oscillations predicts excitability fluctuations in the primary motor cortex as reflected by motor evoked potentials amplitude: an EEG-TMS study. Hum Brain Mapp. (2014) 35:1969–80. 10.1002/hbm.22306 23868714 PMC6869650

[91] Ferreri F Vecchio F Guerra A Miraglia F Ponzo D Vollero L. Age related differences in functional synchronization of EEG activity as evaluated by means of TMS-EEG coregistrations. Neurosci Lett. (2017) 647:141–6. 10.1016/j.neulet.2017.03.021 28323091

[92] Keil J Timm J Sanmiguel I Schulz H Obleser J Schönwiesner M. Cortical brain states and corticospinal synchronization influence TMS-evoked motor potentials. J Neurophysiol. (2014) 111:513–9. 10.1152/jn.00387.2013 24198325

[93] Chung SW Sullivan CM Rogasch NC Hoy KE Bailey NW Cash RFH. The effects of individualised intermittent theta burst stimulation in the prefrontal cortex: a TMS-EEG study. Hum Brain Mapp. (2019) 40:608–27. 10.1002/hbm.24398 30251765 PMC6865598

[94] Desideri D Zrenner C Gordon PC Ziemann U Belardinelli P. Nil effects of μ-rhythm phase-dependent burst-rTMS on cortical excitability in humans: a resting-state EEG and TMS-EEG study. PLoS ONE. (2018) 13:e0208747. 10.1371/journal.pone.0208747 30532205 PMC6286140

[95] Momi D Ozdemir RA Tadayon E Boucher P Di Domenico A Fasolo M. Phase-dependent local brain states determine the impact of image-guided TMS on motor network EEG synchronization. J Physiol. (2021) 600:1455–71. 10.1113/JP282393 34799873 PMC9728936

[96] Zrenner B Zrenner C Gordon PC Belardinelli P McDermott EJ Soekadar SR. Brain oscillation-synchronized stimulation of the left dorsolateral prefrontal cortex in depression using real-time EEG-triggered TMS. Brain Stim. (2020) 13:197–205. 10.1016/j.brs.2019.10.007 31631058

[97] Jin Y Phillips B. A pilot study of the use of EEG-based synchronized Transcranial Magnetic Stimulation (sTMS) for treatment of Major Depression. BMC Psychiatry. (2014) 14:13. 10.1186/1471-244X-14-13 24438321 PMC3904196

[98] Leuchter AF Cook IA Feifel D Goethe JW Husain M Carpenter LL. Efficacy and safety of low-field Synchronized Transcranial Magnetic Stimulation (sTMS) for treatment of major depression. Brain Stimul. (2015) 8:787–94. 10.1016/j.brs.2015.05.005 26143022

[99] Isserles M Rosenberg O Dannon P Levkovitz Y Kotler M Deutsch F. Cognitive-emotional reactivation during deep transcranial magnetic stimulation over the prefrontal cortex of depressive patients affects antidepressant outcome. J Affect Disord. (2011) 128:235–42. 10.1016/j.jad.2010.06.038 20663568

[100] Hendrikse J Kandola A Coxon J Rogasch N Yücel M. Combining aerobic exercise and repetitive transcranial magnetic stimulation to improve brain function in health and disease. Neurosci Biobehav Rev. (2017) 83:11–20. 10.1016/j.neubiorev.2017.09.023 28951250

[101] Yang Y-W Pan W-X Xie Q. Combined effect of repetitive transcranial magnetic stimulation and physical exercise on cortical plasticity. Neural Regen Res. (2020) 15:1986–94. 10.4103/1673-5374.282239 32394946 PMC7716032

[102] Drysdale AT Grosenick L Downar J Dunlop K Mansouri F Meng Y. Resting-state connectivity biomarkers define neurophysiological subtypes of depression. Nat Med. (2017) 23:28–38. 10.1038/nm.4246 27918562 PMC5624035

[103] Cheeran B Talelli P Mori F Koch G Suppa A Edwards M. A common polymorphism in the brain-derived neurotrophic factor gene (BDNF) modulates human cortical plasticity and the response to rTMS. J Physiol. (2008) 586:5717–25. 10.1113/jphysiol.2008.159905 18845611 PMC2655403

[104] Cash RFH Udupa K Gunraj CA Mazzella F Daskalakis ZJ Wong AHC. Influence of BDNF Val66Met polymorphism on excitatory-inhibitory balance and plasticity in human motor cortex. Clin Neurophysiol. (2021) 132:2827–39. 10.1016/j.clinph.2021.07.029 34592560

[105] Shah-Basak P Harvey DY Parchure S Faseyitan O Sacchetti D Ahmed A. Brain-derived neurotrophic factor polymorphism influences response to single-pulse transcranial magnetic stimulation at rest. Neuromodulation. (2020) 24:854–62. 10.1111/ner.13287 33090650 PMC8032803

[106] Chang WH Hwang JM Uhm KE Pascual-Leone A Kim YH. Corticospinal excitability in the non-dominant hand is affected by BDNF genotype. Neurol Sci. (2017) 38:241–7. 10.1007/s10072-016-2749-9 27783184

[107] Jannati A Block G Oberman LM Rotenberg A Pascual-Leone A. Interindividual variability in response to continuous theta-burst stimulation in healthy adults. Clin Neurophysiol. (2017) 128:2268–78. 10.1016/j.clinph.2017.08.023 29028501 PMC5675807

[108] Parchure S Harvey DY Shah-Basak PP DeLoretta L Wurzman R Sacchetti D. Brain-derived neurotrophic factor gene polymorphism predicts response to continuous theta burst stimulation in chronic stroke patients. Neuromodulation. (2021). 10.1111/ner.13495 35667772 PMC8913155

[109] McHughen SA Pearson-Fuhrhop K Ngo VK Cramer SC. Intense training overcomes effects of the Val66Met BDNF polymorphism on short-term plasticity. Exp Brain Res. (2011) 213:415–22. 10.1007/s00221-011-2791-z 21769545

